# Proximal Binding Pocket Arg717 Substitutions in Escherichia coli AcrB Cause Clinically Relevant Divergencies in Resistance Profiles

**DOI:** 10.1128/aac.02392-21

**Published:** 2022-03-21

**Authors:** Martijn Zwama, Kunihiko Nishino

**Affiliations:** a SANKEN (The Institute of Scientific and Industrial Research), Osaka University, Ibaraki, Osaka, Japan; b Graduate School of Pharmaceutical Sciences, Osaka University, Suita, Osaka, Japan; c Center for Infectious Disease Education and Research, Osaka University, Suita, Osaka, Japan

**Keywords:** antimicrobial resistance, multidrug resistance, RND, AcrB, macrolides, fluoroquinolones

## Abstract

Recent mutations in RND efflux pumps in clinical strains have further increased multidrug resistance. We show that R717L and R717Q substitutions (found in azithromycin-resistant Salmonella enterica spp.) in the Escherichia coli efflux pump AcrB dramatically increase macrolide, as well as fluoroquinolone, resistance. On the other hand, cells became more susceptible to novobiocin and β-lactam cloxacillin. We urge the control of, and adjustments to, treatments with antibiotics and the need for novel antibiotics and efflux pump inhibitors.

## INTRODUCTION

**FIG 1:**
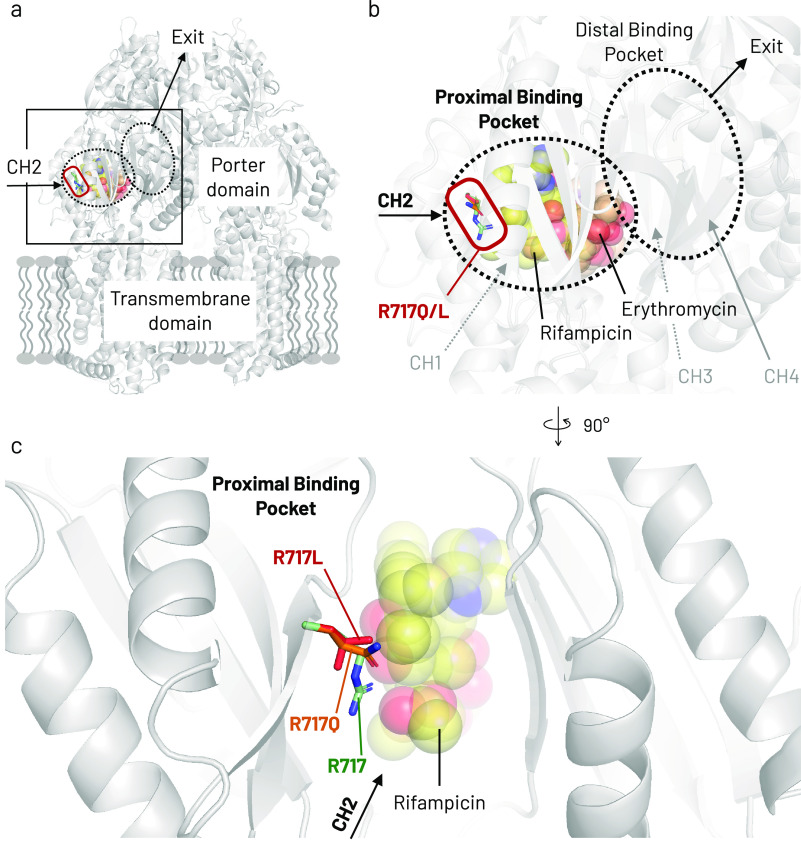
Location of Arg717 and the R717Q/L amino acid substitutions in AcrB-Ec. (a) Side view of the entire AcrB-Ec trimer. (b) A close-up view of the rectangle area highlighted in panel a. Two drug molecules are present in the proximal binding pocket (rifampicin and erythromycin). The Arg717 residue and the R717Q/L substitutions are located at the entrance of the pocket. (c) Close-up inwards view of the proximal binding pocket and Arg717 area. Rifampicin can be seen behind the Arg717 residue and the R717Q/L substitutions. CH1–4, Channel 1–4. Colors: red box, substitution area; yellow spheres, rifampicin; orange spheres, erythromycin; green sticks, Arg717; orange sticks, R717Q; red sticks, R717L. PDB accession codes: 4DX5 (for the main structure and the mutagenesis); 3AOC (for the erythromycin coordinates); 3AOD (for the rifampicin and the wild-type Arg717 side chain coordinates).

Multidrug-resistant pathogens resist multiple antibiotics (1–4), which can be caused by over-expressed intrinsic and acquired (5–7) efflux pumps (5, 8, 9). In Gram-negative bacteria, RND-type efflux pumps (7, 10) attribute to multidrug resistance (MDR) by expelling structurally unrelated antibiotics (11). Recently, amino acid substitutions further increased resistance (12). Substitutions in the proximal binding pocket (PBP, Fig. 1) (11–14) of Salmonella enterica Serovars Typhi and Paratyphi A pump AcrB caused azithromycin (AZM)-resistant phenotypes (12). This is concerning, as AZM is often the last available typhoid treatment option (15, 16). In 2010, AZM-resistant *S.* serovars Paratyphi A strains from Pakistan were first reported (17). The AZM breakpoint has been determined to be >16 μg/mL for S. Typhi. (18). Hooda et al. (2019) first described R717Q/L substitutions in AcrB from Salmonella isolates from Bangladesh. Multiple strains harboring R717Q/L showed AZM resistance between 32–64 μg/mL (19). R717Q/L were also found in AZM-resistant S. Typhi strains from Nepal (15), Pakistan (16), and India (20). Phylogenetic analysis showed the R717Q mutation likely spontaneously and independently emerged (16). Sajib et al. (2021) showed that most AZM-resistant Typhi and Paratyphi A strains from Bangladesh had these substitutions. They predicted that the first Arg717 substitutions emerged around 2010 and that a travel-related R717Q mutant was also found in the United Kingdom (21). Additionally, after analysis of a similar R714G substitution in MtrD from Neisseria gonorrhoeae (22), different substitutions (R714C/H/L) were found in some AZM-resistant isolates (23). Here, we investigated R717Q/L in Escherichia coli AcrB (AcrB-Ec), closely related to Salmonella AcrB, expressed in E. coli. We compared the effects to other clinical mutations, namely, K823E/N (causing enhanced AZM resistance by MtrD) (22–25) and G288D (causing fluoroquinolone resistance in a S. Typhimurium clinical strain) (26).

S. enterica (Typhi and Paratyphi A) AcrB and AcrB-Ec comprise 1,049 amino acids (Fig. S1 in the supplemental material) and share about 94% identity (Fig. S2–S4) (27). R717Q/L side chains are shorter and more hydrophobic than Arg717 (Fig. 1c, S5). Mutant and wild-type AcrB-Ec were equally expressed (Fig. S6). Table 1 shows the agar-plate (solid) MIC results of 20 compounds. Compared to wild-type AcrB, R717Q/L give divergent resistance spectra. MICs generally increased for macrolides and fluoroquinolones but decreased for novobiocin (NOV) and β-lactam cloxacillin (CLX). For clarithromycin (CLR) and erythromycin (ERY), the MICs of R717Q/L were >256 and 256 μg/mL, respectively, compared to 128 μg/mL for the wild type. The MIC for AZM increased significantly by 4- and 8-fold for R717Q and R717L (64 and 128 μg/mL, compared to 16 μg/mL for wild-type AcrB). Mutant cells grew as full and healthy colonies on the agar plates at their highest viable macrolide concentrations. Ofloxacin (OFX), levofloxacin (LVX), and moxifloxacin (MXF) MICs increased 2-fold. For R717Q, the monobactam aztreonam (ATM) MIC increased by 4-fold. R717L increased the ethidium bromide (EtBr) MIC 2-fold. Interestingly, R717Q and R717L both caused 2-fold NOV and CLX MIC decreases.

**TABLE 1:** Antimicrobial susceptibility of AcrB-Ec expressing cells with several amino acid substitutions^*a*^

	Minimum inhibitory concn (MIC) (μg mL^−1^)^*b*^																			
	Planar aromatic cations					Macrolides			β-lactams		Quinolones						Other		Bile salts	
Strain^*c*^	EtBr	MtB	ACR	CV	BZK	ERY	AZM	CLR	CLX	ATM	NAL	NOR	CIP	OFX	LVX	MXF	NOV	MIN	Mix	DEOX
Vector	16	16	16	2	4	2	<0.5	2	1	1/16	1	1/128	1/256	1/64	1/128	1/256	4	0.125	2,500	625
WT	512	>1,024	256	8–16	128	128	16	128	256	1/32	4	1/32	1/64	1/16	1/32	1/16	256	1	>20,000	10,000
R717Q	512	>1,024	256	8	128	**256**	**64**	**>256**	*128*	**0.125**	4	1/32	1/64	**0.125**	**1/16**	**0.125**	*128*	1	>20,000	10,000
R717L	**1,024**	>1,024	256	8	128	**256**	**128**	**>256**	*128*	1/32	4	1/32	1/64	**0.125**	1/32	**0.125**	*128*	1	>20,000	10,000
G288D	*256*	*1,024*	*128*	8	*64*	*64*	*4*	*64*	*128*	**0.125**	4	1/32	1/64	1/16	1/32	1/16	*64*	1	>20,000	**20,000**
E826K	512	>1,024	*128*	8	128	128	*4*	*64*–128	256	**1/16**	4	1/32	1/64	1/16	1/32	1/16	256	1	>20,000	10,000
E826N	512	>1,024	256	8	128	128	16	128	256	**1/16**	4	1/32	1/64	1/16	1/32	1/16	256	1	>20,000	**20,000**

a EtBr, ethidium bromide; MtB, methylene blue; ACR, acriflavine; CV, crystal violet; BZK, benzalkonium; ERY, erythromycin; AZM, azithromycin; CLR, clarithromycin; CLX, cloxacillin; ATM, aztreonam; NAL, nalidixic acid; NOR, norfloxacin; CIP, ciprofloxacin; OFX, ofloxacin; LVX, levofloxacin; MXF, moxifloxacin; NOV, novobiocin; MIN, minocycline; Mix, bile sale mixture; DEOX, deoxycholate; WT, wild-type AcrB-Ec.

b His-tagged wild-type AcrB-Ec and mutant AcrB-Ec were expressed from pBAD33 plasmids and expressed in MG1655Δ*acrB* cells, MIC values determined by serial dilution agar plates. Bold indicates a 2-fold increase in MIC, bold underlined indicates a 4-fold or more increase in MIC, and italic indicates a decrease of 2-fold or more in MIC compared to wild-type.

c Strain information: all strains are Escherichia coli MG1655Δ*acrB* with different plasmids, namely Vector, pBAD33; WT, pBAD33acrBhis; rest, pBAD33acrBhis with the respective amino acid substitution.

To compare the R717Q/L mutations, we introduced other clinical substitutions in AcrB-Ec, found in N. gonorrhoeae MtrD and S. Typhimurium AcrB. In MtrD, K823E/N were observed in macrolide-resistant strains (22–25). In AcrB, G288D conferred increased fluoroquinolone resistance in a clinical strain (26). Therefore, we introduced the substitutions G288D, E826K, and E826N in AcrB-Ec. Lys823 in MtrD corresponds to Glu826 in AcrB-Ec (Fig. S7); thus, we introduced the K823E “reversed” substitution in AcrB-Ec. This residue lies deeper in the PBP “behind” Arg717 (13, 22). Gly288 is a distal binding pocket residue (Fig. 1b), close to the hydrophobic pit, where multiple drugs bind in the binding monomer (13, 22, 28–33). Results are shown in Table 1. G288D decreased most planar aromatic cation (PAC), macrolide, β-lactam CLX, and NOV MICs. Contrarily, G288D increased ATM and deoxycholate (DEOX) MICs by 4- and 2-fold, respectively. E826K increased susceptibility for acriflavine (ACR) and AZM (and slightly for CLR); however, MICs were 2-fold higher for ATM. E826N MICs for ATM and DEOX increased by 2-fold. The increased susceptibility of AcrB-Ec(E826K) cells to ACR, AZM, and CLR could correspond with the decreased susceptibility to AZM (23) and ERY (22, 24) for strains harboring the mutated MtrD(K823E).

**FIG 2:**
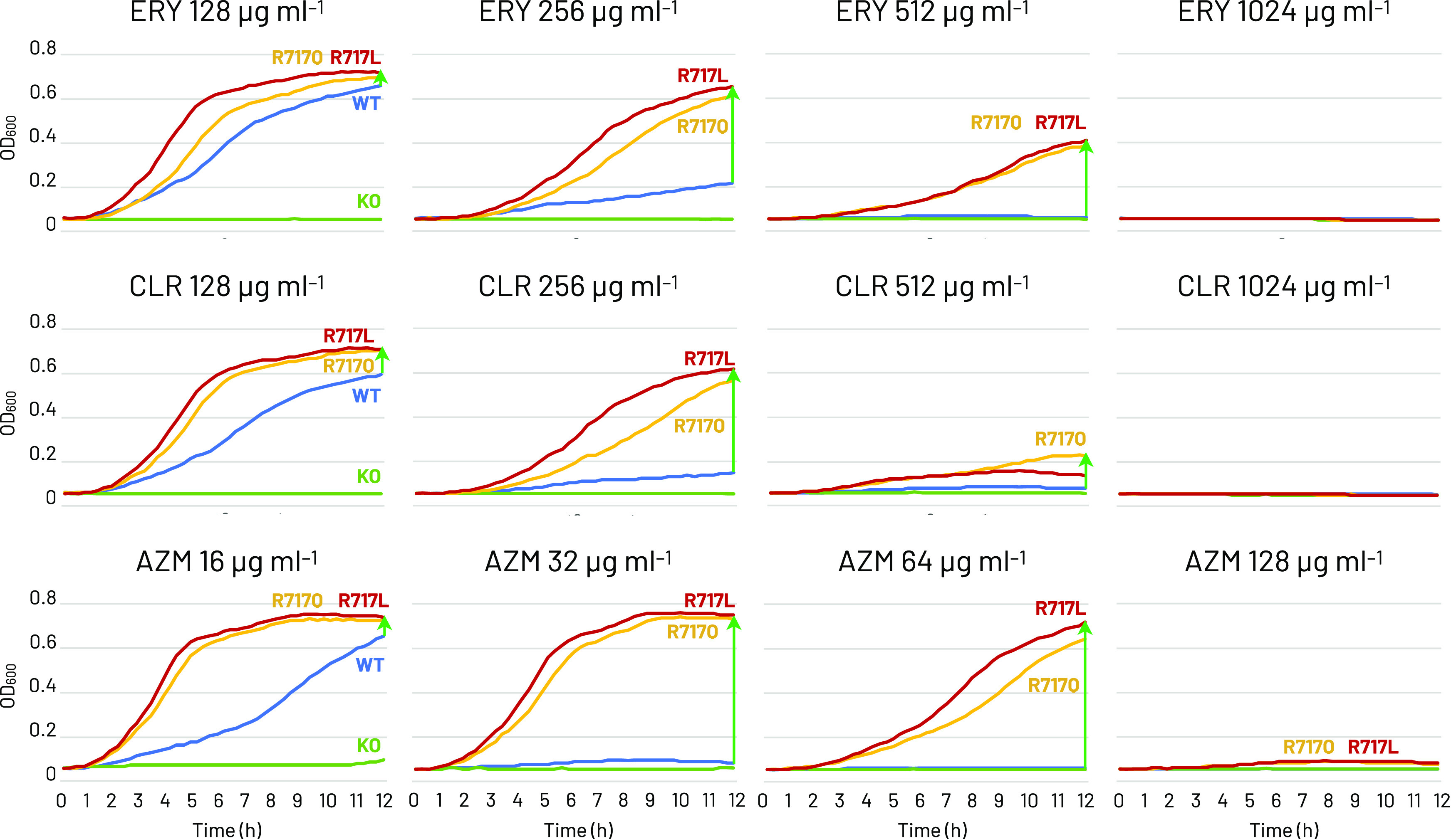
Growth ability of E. coli MG1655Δ*acrB* cells expressing wild-type, R717Q, and R717L mutant AcrB-Ec under several macrolide concentrations. Top lane, growth ability under erythromycin; middle lane, under clarithromycin; bottom lane, under azithromycin. Blue, yellow, red, and green indicate wild-type AcrB, R717Q mutant, R717L mutant, and vector only, respectively. The green arrow mark indicates the increase in growth ability of the mutant strains compared to the wild-type strain. ERY, erythromycin; CLR, clarithromycin; AZM, azithromycin. Experiments repeated twice provided similar results; shown is one of the results.

**FIG 3:**
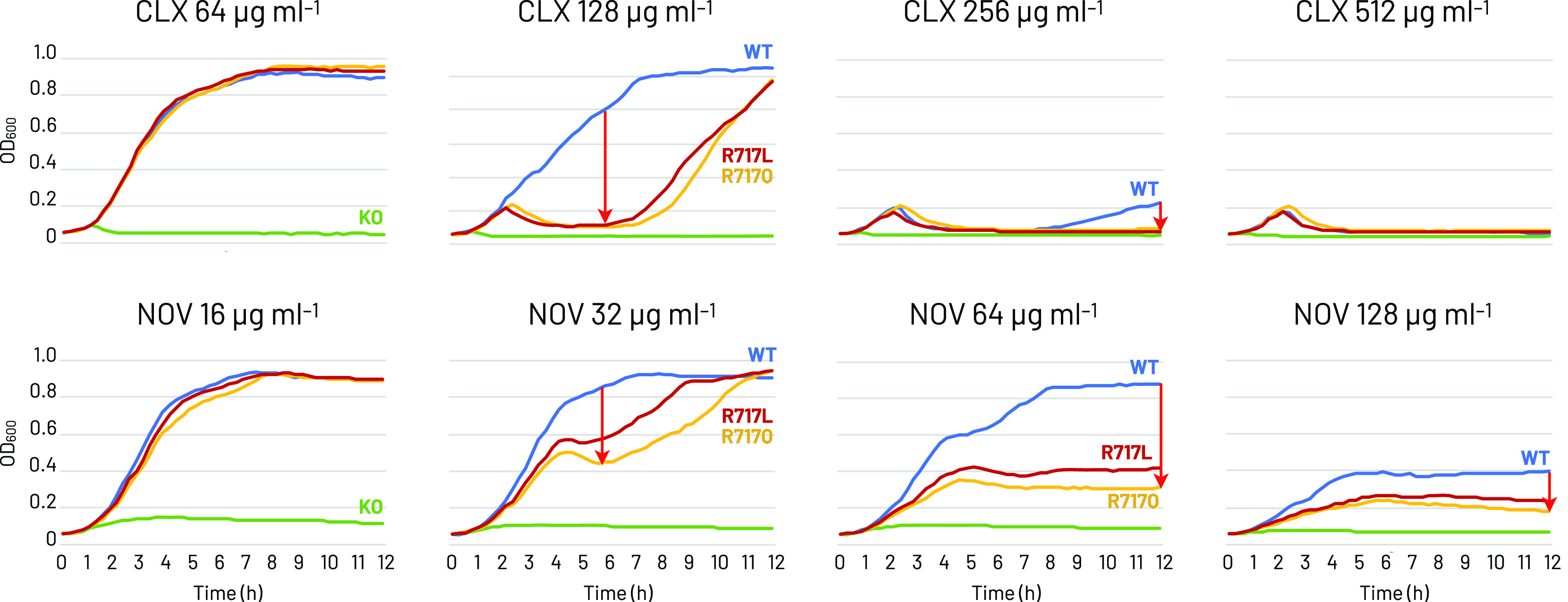
Growth ability of E. coli MG1655Δ*acrB* cells expressing wild-type, R717Q, and R717L mutant AcrB-Ec under cloxacillin and novobiocin. Top lane, growth ability under cloxacillin; bottom lane, under novobiocin. Blue, yellow, red, and green indicate wild-type AcrB, R717Q mutant, R717L mutant, and vector only, respectively. The red arrow mark indicates the decrease in growth ability of the mutant strains compared to the wild-type strain. CLX, cloxacillin; NOV, novobiocin. Experiments repeated twice provided similar results; shown is one of the results.

Solid plate MICs (Table 1) showed an up to 8-fold increase in macrolide MICs for R717Q/L mutants. Furthermore, clinically relevant G288D generally caused decreased MICs. The reversed substitution E826K decreased multiple drugs’ MICs. Thus, the 8-fold AZM, the 2-fold ERY, and the ≥4-fold CLR MIC increases show a significant macrolide export gain-of-function for AcrB-Ec by the R717Q/L substitutions. Therefore, we checked the growth of R717Q/L cells in macrolide, CLX, and NOV supplemented liquid medium. Fig. 2 (macrolides) shows that wild-type cell growth is significantly inhibited at 256 μg/mL and completely inhibited at 512 μg/mL ERY (top lane). Contrarily, R717Q/L mutants grow fully at 256 μg/mL and significantly grow even under 512 μg/mL. Similarly, for CLR (middle lane), wild-type cell growth is inhibited at 256 μg/mL, while mutant cells can grow fully. At 512 μg/mL, mutant cells still slightly grow. For AZM (bottom lane), wild-type cell growth was already partly inhibited at 16 μg/mL and fully at 32 μg/mL. Contrarily, mutants could fully grow, even in 64 μg/mL (even in 128 μg/mL, the mutants seem very slightly viable). These results corroborate the significant resistance increase caused by R717Q/L for macrolides, especially AZM. Under all conditions, R717L seemed somewhat more viable than R717Q. Fig. 3 shows inhibited cell growth for R717Q/L, compared to the wild type, in CLX and NOV supplemented medium, corroborating the plate MIC results (Table 1).

**FIG 4:**
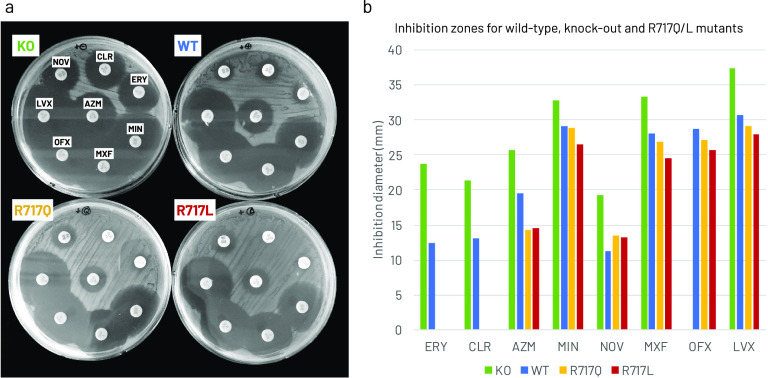
Kirby-Bauer disk diffusion susceptibility testing of E. coli MG1655Δ*acrB* cells expressing wild-type, R717Q, and R717L mutant AcrB-Ec under various antibiotics. (a) Grayscale images of knockout, wild-type, and mutant E. coli grown on Mueller-Hilton agar plates, supplemented with resazurin and arabinose. Disks with specific antibiotics of interest can be seen on all four plates, along with the growth inhibition zones around the disks. (b) Quantification of the inhibition zones. The vertical axis shows the inhibition diameter in mm. Growth of the cells up to the antibiotic disk is denoted as 0 mm. (a–b) Blue, yellow, red, and green indicate wild-type AcrB, R717Q mutant, R717L mutant, and vector only, respectively. KO, *acrB* knockout (vector only); WT, wild-type; ERY, erythromycin; CLR, clarithromycin; AZM, azithromycin; NOV, novobiocin; LVX, levofloxacin; OFX, ofloxacin; MXF, moxifloxacin; MIN, minocycline. Shown is one of the results; repeats gave similar results.

To further check the clinical significance, we performed Kirby-Bauer disk diffusion susceptibility tests. Table 1 shows R717Q/L also increased fluoroquinolone resistance, besides increased macrolide resistance. S. Typhi strains from Pakistan and Nepal show that R717Q/L cause reduced fluoroquinolone susceptibility; however, they note a double mutation in *gyrA* contributes to this phenotype (15, 16). Nonetheless, we wanted to further investigate the consequences of R717Q/L in AcrB-Ec on fluoroquinolone and macrolide resistance. Fig. 4 shows the disk diffusion susceptibility results for macrolides, fluoroquinolones, NOV, and minocycline (MIN). When AcrB-Ec is expressed, the inhibition zones for all compounds decrease (Fig. 4, WT versus KO). R717Q/L mutations significantly decrease the inhibition zones further for all macrolides and fluoroquinolones (visible by the naked eye in Fig. 4a, quantified in Fig. 4b). R717L has slightly more impact on the inhibition zone decreases than R717Q (Fig. 4b). Interestingly, R717L also decreases the inhibition zone for MIN. Similar to Table 1 and Fig. 3, R717Q/L increased NOV susceptibility (Fig. 4a and b).

**FIG 5:**
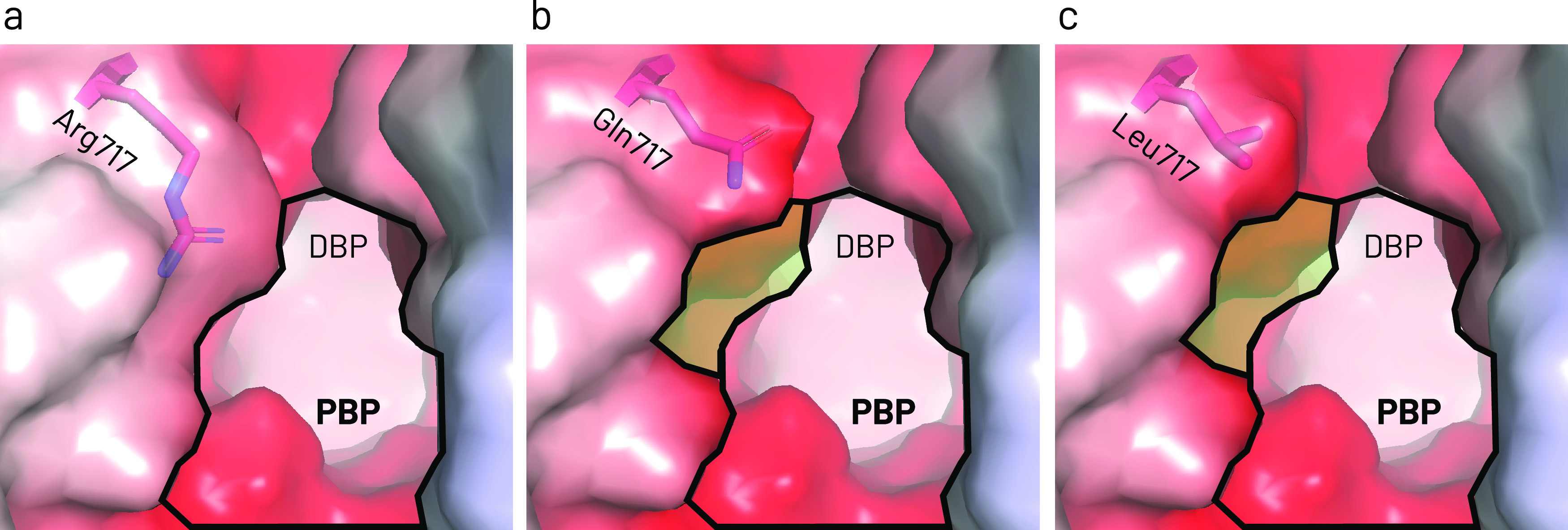
Comparison of the entrance area of the proximal binding pocket in the Access (or Loose [L]) monomer of AcrB-Ec for wild-type and the mutants. (a) Wild-type, (b) R717Q mutant, and (c) R717L mutant AcrB-Ec. (a–c) The residues of interest are depicted as pink sticks. The electrostatic surface is depicted from red to blue. The bold lines indicate the wild-type AcrB-Ec proximal binding pocket entrance area at the Arg717 location. In (b) and (c), the green highlighted area depicts the increased area for R717Q (Gln717) and R717L (Leu717) mutant AcrB-Ec, respectively. These images show the entrance of the proximal binding pocket, leading to the distal binding pocket in the background. PBP, proximal binding pocket; DBP, distal binding pocket. PDB accession code: 4DX5.

We showed that R717Q/L substitutions in AcrB-Ec confer significant increased macrolides resistance, with an up to 8-fold increase in MICs. These findings corroborate the phenotypes of AZM-resistant S. Typhi and Paratyphi A strains harboring the R717Q/L mutations (19–21). Interestingly, R717Q/L caused a 2-fold MIC decrease for CLX and NOV. This could (partly) explain the susceptibility of AZM-resistant S. Typhi R717Q strains from Pakistan, which were still susceptible to third-generation cephalosporins (16). According to the EUCAST database, the theoretical AZM epidemiological cut-off value for E. coli is 16 μg/mL (34). Our wild-type E. coli MG1655 strain has an AZM plate MIC of 16 μg/mL, and R717Q/L mutants 64 and 128 μg/mL, respectively (Table 1). Mutants also have an AZM liquid MIC of 128 μg/mL (Fig. 2). These substitutions caused a hydrophilicity decrease at the entrance of the PBP (Fig. 1, 5, S5). Gln is polar while Leu is hydrophobic; still, both substitutions cause macrolide MIC increases. Macrolides are hydrophobic molecules; thus, the hydrophilicity decrease (and the removal of a positive charge) partly explains the increased resistance. Additionally, the side chains are significantly shorter for R717Q/L, enlarging the PBP entrance (Fig. 1c, Fig. 5), further explaining the enhanced efflux of bulky macrolides (13). We showed that bulky Trp substitutions at the PBP entrance decreased ERY MICs significantly (14), corroborating the impact of space-limiting substitutions on macrolide-export. The increased space and the increased hydrophobicity can explain why R717L seems slightly more active in exporting macrolides than R717Q (Table 1, Fig. 2). Furthermore, the inhibition zone for R717L was also significantly smaller than for R717Q for fluoroquinolones and MIN (Fig. 4). Basically no difference was found between the mutants on the growth under CLX, while R717L seemed slightly more viable than R717Q under NOV (Fig. 3).

As explained, besides increased macrolide and fluoroquinolone resistance, we observed decreased MICs for CLX and NOV. Therefore, it may be clinically interesting to combine multiple antibiotics to treat typhoid and paratyphoid fever to mitigate resistance and enhance treatment. For example, a combination of β-lactams and AZM may enhance the treatment of Salmonella infections. Additionally, we observed this significant gain-of-function in E. coli AcrB for the first time, showing that these mutations in other pathogenic bacteria may significantly affect clinical treatment options. These results further imply the importance of adjusted antibiotics treatments, and the need for novel antibiotics and efflux pump inhibitors.

E. coli MG1655 (35) was used to create Δ*acrB* (NKE96) (36) by gene deletion (37). Bacterial strains were grown at 37°C in Luria-Bertani broth (38). His-tagged AcrB was expressed from pBAD33acrBhis, and point mutations were introduced by PCR (GenScript). Susceptibility testing in liquid and on solid media was determined by adding the toxic compounds by serial dilutions. Cell cultures (supplemented with 10 mM arabinose) were incubated until OD_600 nm_ 0.6 and diluted to 0.05. For liquid growth curves, OD_600 nm_ readings were performed. For LB agar experiments, cells were stamped on agar plates and incubated overnight. Disk diffusion susceptibility was performed according to (39, 40), with slight modifications. In short: Mueller-Hinton agar plates were supplemented with resazurin (41). Overnight cultures were diluted and grown until OD_600 nm_ 0.6, and then diluted to OD_600 nm_ 0.1 with PBS buffer. Cells were streaked, and antibiotic discs were added. Plates were left overnight at 37°C (42).

### Data availability.

Data are available in the published article itself, and the supporting figures and tables are available as supplemental material. Other data that support the findings of this study are available from the corresponding authors upon request.
