# Supplementary material for: Proximal Binding Pocket Arg717 Substitutions in Escherichia coli AcrB Cause Clinically Relevant Divergencies in Resistance Profiles
Source: Antimicrob Agents Chemother. 2022 Mar 21;66(4):e02392-21. doi: 10.1128/aac.02392-21 (PMC9017336; doi:10.1128/aac.02392-21)
Supplement: Supplemental file 1 — Supplemental material. Download aac.02392-21-s0001.pdf, PDF file, 4.5 MB [file aac.02392-21-s0001.pdf]

```
AcrB_Ec_MG1655                        MPNFFIDRPIFAWVIAIIIMLAGGLAILKLPVAQYPTIAPPAVTISASYPGADAKTVQDT          60
AcrB_Sa_Salmonella_Paratyphi_A        MPNFFIDRPIFAWVIAIIIMLAGGLAIFKLPVAQYPTIAPPAVTISATYPGADAKTVQDT          60
AcrB_Sa_Salmonella_enterica           MPNFFIDRPIFAWVIAIIIMLAGGLAILKLPVAQYPTIAPPAVTISATYPGADAKTVQDT          60
AcrB_Sa_Salmonella_Typhi              MPNFFIDRPIFAWVIAIIIMLAGGLAILKLPVAQYPTIAPPAVTISATYPGADAKTVQDT          60
                                      ***************************:*******************:************

AcrB_Ec_MG1655                        VTQVIEQNMNGIDNLMYMSSNSDSTGTVQITLTFESGTDADIAQVQVQNKLQLAMPLLPQ          120
AcrB_Sa_Salmonella_Paratyphi_A        VTQVIEQNMNGIDNLMYMSSNSDSTGTVQITLTFESGTDADIAQVQVQNKLQLAMPLLPQ          120
AcrB_Sa_Salmonella_enterica           VTQVIEQNMNGIDNLMYMSSNSDSTGTVQITLTFESGTDADIAQVQVQNKLQLAMPLLPQ          120
AcrB_Sa_Salmonella_Typhi              VTQVIEQNMNGIDNLMYMSSNSDSTGTVQITLTFESGTDADIAQVQVQNKLQLAMPLLPQ          120
                                      ************************************************************

AcrB_Ec_MG1655                        EVQQQGVSVEKSSSSFLMVVGVINTDGTMTQEDISDYVAANMKDAISRTSGVGDVQLFGS          180
AcrB_Sa_Salmonella_Paratyphi_A        EVQQQGVSVEKSSSSFLMVVGVINTDGTMTQEDISDYVAANMKDPISRTSGVGDVQLFGS          180
AcrB_Sa_Salmonella_enterica           EVQQQGVSVEKSSSSFLMVVGVINTDGTMTQEDISDYVAANMKDPISRTSGVGDVQLFGS          180
AcrB_Sa_Salmonella_Typhi              EVQQQGVSVEKSSSSFLMVVGVINTDGTMTQEDISDYVAANMKDPISRTSGVGDVQLFGS          180
                                      ******************************************** ***************

AcrB_Ec_MG1655                        QYAMRIWMNPNELNKFQLTPVDVITAIKAQNAQVAAGQLGGTPPVKGQQLNASIIAQTRL          240
AcrB_Sa_Salmonella_Paratyphi_A        QYAMRIWMNPTELTKYQLTPVDVINAIKAQNAQVAAGQLGGTPPVKGQQLNASIIAQTRL          240
AcrB_Sa_Salmonella_enterica           QYAMRIWMNPTELTKYQLTPVDVINAIKAQNAQVAAGQLGGTPPVKGQQLNASIIAQTRL          240
AcrB_Sa_Salmonella_Typhi              QYAMRIWMNPTELTKYQLTPVDVINAIKAQNAQVAAGQLGGTPPVKGQQLNASIIAQTRL          240
                                      **********.**.*:********.***********************************

AcrB_Ec_MG1655                        TSTEEFGKILLKVNQDGSRVLLRDVAKIELGGENYDIIAEFNGQPASGLGIKLATGANAL          300
AcrB_Sa_Salmonella_Paratyphi_A        TSTDEFGKILLKVNQDGSQVRLRDVAKIELGGENYDVIAKFNGQPASGLGIKLATGANAL          300
AcrB_Sa_Salmonella_enterica           TSTDEFGKILLKVNQDGSQVRLRDVAKIELGGENYDVIAKFNGQPASGLGIKLATGANAL          300
AcrB_Sa_Salmonella_Typhi              TSTDEFGKILLKVNQDGSQVRLRDVAKIELGGENYDVIAKFNGQPASGLGIKLATGANAL          300
                                      ***:**************:* ***************:**:********************

AcrB_Ec_MG1655                        DTAAAIRAELAKMEPFFPSGLKIVYPYDTTPFVKISIHEVVKTLVEAIILVFLVMYLFLQ          360
AcrB_Sa_Salmonella_Paratyphi_A        DTATAIRAELKKMEPFFPPGMKIVYPYDTTPFVKISIHEVVKTLVEAIILVFLVMYLFLQ          360
AcrB_Sa_Salmonella_enterica           DTATAIRAELKKMEPFFPPGMKIVYPYDTTPFVKISIHEVVKTLVEAIILVFLVMYLFLQ          360
AcrB_Sa_Salmonella_Typhi              DTATAIRAELKKMEPFFPPGMKIVYPYDTTPFVKISIHEVVKTLVEAIILVFLVMYLFLQ          360
                                      ***:****** ******* *:***************************************

AcrB_Ec_MG1655                        NFRATLIPTIAVPVVLLGTFAVLAAFGFSINTLTMFGMVLAIGLLVDDAIVVVENVERVM          420
AcrB_Sa_Salmonella_Paratyphi_A        NFRATLIPTIAVPVVLLGTFAVLAAFGFSINTLTMFGMVLAIGLLVDDAIVVVENVERVM          420
AcrB_Sa_Salmonella_enterica           NFRATLIPTIAVPVVLLGTFAVLAAFGFSINTLTMFGMVLAIGLLVDDAIVVVENVERVM          420
AcrB_Sa_Salmonella_Typhi              NFRATLIPTIAVPVVLLGTFAVLAAFGFSINTLTMFGMVLAIGLLVDDAIVVVENVERVM          420
                                      ************************************************************

AcrB_Ec_MG1655                        AEEGLPPKEATRKSMGQIQGALVGIAMVLSAVFVPMAFFGGSTGAIYRQFSITIVSAMAL          480
AcrB_Sa_Salmonella_Paratyphi_A        TEEGLPPKEATRKSMGQIQGALVGIAMVLSAVFIPMAFFGGSTGAIYRQFSITIVSAMAL          480
AcrB_Sa_Salmonella_enterica           TEEGLPPKEATRKSMGQIQGALVGIAMVLSAIFIPMAFFGGSTGAIYRQFSITIVSAMAL          480
AcrB_Sa_Salmonella_Typhi              TEEGLPPKEATRKSMGQIQGALVGIAMVLSAVFIPMAFFGGSTGAIYRQFSITIVSAMAL          480
                                      :******************************:*:**************************

AcrB_Ec_MG1655                        SVLVALILTPALCATMLKPIAKGDHGEGKKGFFGWFNRMFEKSTHHYTDSVGGILRSTGR          540
AcrB_Sa_Salmonella_Paratyphi_A        SVLVALILTPALCATMLKPVAKGDHGEGKKGFFGWFNRLFDKSTHHYTDSVGNILRSTGR          540
AcrB_Sa_Salmonella_enterica           SVLVALILTPALCATMLKPVAKGDHGEGKKGFFGWFNRLFDKSTHHYTDSVGNILRSTGR          540
AcrB_Sa_Salmonella_Typhi              SVLVALILTPALCATMLKPVAKGDHGEGKKGFFGWFNRLFDKSTHHYTDSVGNILRSTGR          540
                                      *******************:******************:*:***********.*******

AcrB_Ec_MG1655                        YLVLYLIIVVGMAYLFVRLPSSFLPDEDQGVFMTMVQLPAGATQERTQKVLNEVTHYYLT          600
AcrB_Sa_Salmonella_Paratyphi_A        YLLLYLIIVVGMAYLFVRLPSSFLPDADQGVFLTMVQLPAGATQERTQKVLDEVTDYYLN          600
AcrB_Sa_Salmonella_enterica           YLLLYLIIVVGMAYLFVRLPSSFLPDEDQGVFLTMVQLPAGATQERTQKVLDEVTDYYLN          600
AcrB_Sa_Salmonella_Typhi              YLLLYLIIVVGMAYLFVRLPSSFLPDEDQGVFLTMVQLPAGATQERTQKVLDEVTDYYLN          600
                                      **:*********************** *****:******************:***.***.

AcrB_Ec_MG1655                        KEKNNVESVFAVNGFGFAGRGQNTGIAFVSLKDWADRPGEENKVEAITMRATRAFSQIKD          660
AcrB_Sa_Salmonella_Paratyphi_A        KEKANVESVFAVNGFGFAGRGQNTGIAFVSLKDWADRPGEKNKVEAITQRATAAFSQIKD          660
AcrB_Sa_Salmonella_enterica           KEKANVESVFAVNGFGFAGRGQNTGIAFVSLKDWADRPGEKNKVEAITQRATAAFSQIKD          660
AcrB_Sa_Salmonella_Typhi              KEKANVESVFAVNGFGFAGRGQNTGIAFVSLKDWADRPGEKNKVEAITQRATAAFSQIKD          660
                                      *** ************************************:******* *** *******

AcrB_Ec_MG1655                        AMVFAFNLPAIVELGTATGFDFELIDQAGLGHEKLTQARNQLLAEAAKHPDMLTSVRPNG          720
AcrB_Sa_Salmonella_Paratyphi_A        AMVFAFNLPAIVELGTATGFDFELIDQAGLGHEKLTQARNQLFGEVAKYPDLLVGVRPNG          720
AcrB_Sa_Salmonella_enterica           AMVFAFNLPAIVELGTATGFDFELIDQAGLGHEKLTQARNQLFGEVAKYPDLLVGVRPNG          720
AcrB_Sa_Salmonella_Typhi              AMVFAFNLPAIVELGTATGFDFELIDQAGLGHEKLTQARNQLFGEVAKYPDLLVGVRPNG          720
                                      ******************************************:.*.**:**:*..*****

AcrB_Ec_MG1655                        LEDTPQFKIDIDQEKAQALGVSINDINTTLGAAWGGSYVNDFIDRGRVKKVYVMSEAKYR          780
AcrB_Sa_Salmonella_Paratyphi_A        LEDTPQFKIDIDQEKAQALGVSISDINTTLGAAWGGSYVNDFIDRGRVKKVYVMSEAKYR          780
AcrB_Sa_Salmonella_enterica           LEDTPQFKIDIDQEKAQALGVSISDINTTLGAAWGGSYVNDFIDRGRVKKVYVMSEAKYR          780
AcrB_Sa_Salmonella_Typhi              LEDTPQFKIDIDQEKAQALGVSISDINTTLGAAWGGSYVNDFIDRGRVKKVYVMSEAKYR          780
                                      ***********************.************************************

AcrB_Ec_MG1655                        MLPDDIGDWYVRAADGQMVPFSAFSSSRWEYGSPRLERYNGLPSMEILGQAAPGKSTGEA          840
AcrB_Sa_Salmonella_Paratyphi_A        MLPDDINDWYVRGSDGQMVPFSAFSSSRWEYGSPRLERYNGLPSMEILGQAAPGKSTGEA          840
AcrB_Sa_Salmonella_enterica           MLPDDINDWYVRGSDGQMVPFSAFSSSRWEYGSPRLERYNGLPSMEILGQAAPGKSTGEA          840
AcrB_Sa_Salmonella_Typhi              MLPDDINDWYVRGSDGQMVPFSAFSSSRWEYGSPRLERYNGLPSMEILGQAAPGKSTGEA          840
                                      ******.*****.:**********************************************

AcrB_Ec_MG1655                        MELMEQLASKLPTGVGYDWTGMSYQERLSGNQAPSLYAISLIVVFLCLAALYESWSIPFS          900
AcrB_Sa_Salmonella_Paratyphi_A        MAMMEELASKLPSGIGYDWTGMSYQERLSGNQAPALYAISLIVVFLCLAALYESWSIPFS          900
AcrB_Sa_Salmonella_enterica           MAMMEELASKLPSGIGYDWTGMSYQERLSGNQAPALYAISLIVVFLCLAALYESWSIPFS          900
AcrB_Sa_Salmonella_Typhi              MAMMEELASKLPSGIGYDWTGMSYQERLSGNQAPALYAISLIVVFLCLAALYESWSIPFS          900
                                      * :**:******:*:*******************:*************************

AcrB_Ec_MG1655                        VMLVVPLGVIGALLAATFRGLTNDVYFQVGLLTTIGLSAKNAILIVEFAKDLMDKEGKGL          960
AcrB_Sa_Salmonella_Paratyphi_A        VMLVVPLGVIGALLAATFRGLTNDVYFQVGLLTTIGLSAKNAILIVEFAKDLMDKEGKGL          960
AcrB_Sa_Salmonella_enterica           VMLVVPLGVIGALLAATFRGLTNDVYFQVGLLTTIGLSAKNAILIVEFAKDLMDKEGKGL          960
AcrB_Sa_Salmonella_Typhi              VMLVVPLGVIGALLAATFRGLTNDVYFQVGLLTTIGLSAKNAILIVEFAKDLMDKEGKGL          960
                                      ************************************************************

AcrB_Ec_MG1655                        IEATLDAVRMRLRPILMTSLAFILGVMPLVISTGAGSGAQNAVGTGVMGGMVTATVLAIF          1020
AcrB_Sa_Salmonella_Paratyphi_A        VEATLEAVRMRLRPILMTSLAFMLGVMPLVISSGAGSGAQNAVGTGVLGGMVTATVLAIF          1020
AcrB_Sa_Salmonella_enterica           VEAMLEAVRMRLRPILMTSLAFMLGVMPLVISSGAGSGAQNAVGTGVLGGMVTATVLAIF          1020
AcrB_Sa_Salmonella_Typhi              VEAMLEAVRMRLRPILMTSLAFMLGVMPLVISSGAGSGAQNAVGTGVLGGMVTATVLAIF          1020
                                      :** *:****************:*********:**************:************

AcrB_Ec_MG1655                        FVPVFFVVVRRRFSRKNEDIEHSHTVDHH       1049
AcrB_Sa_Salmonella_Paratyphi_A        FVPVFFVVVRRRFSRKSEDIEHSHSTEHR       1049
AcrB_Sa_Salmonella_enterica           FVPVFFVVVRRRFSRKSEDIEHSHSTEHR       1049
AcrB_Sa_Salmonella_Typhi              FVPVFFVVVRRRFSRKSEDIEHSHSTEHR       1049
                                      ****************.*******:.:*:
```

Figure S1. Multiple sequence alignment of four AcrB-type efflux pumps. *Escherichia coli* AcrB from *Escherichia coli* str. K-12 substr. MG1655 (NCBI Reference Sequence: NP_414995.1) and *Salmonella* AcrBs from *Salmonella enterica* subsp. *enterica* serovar Paratyphi A (NCBI Reference Sequence: SUH78479.1), *Salmonella enterica* (NCBI Reference Sequence: WP_109350990.1), and *Salmonella enterica* subsp. *enterica* serovar Typhi (NCBI Reference Sequence: WP_109350990.1). Alignment performed using Clustal Omega. Substitutions are highlighted in yellow.

```
S. ent  1     MPNFFIDRPIFAWVIAIIIMLAGGLAILKLPVAQYPTIAPPAVTISATYPGADAKTVQDT  60
              MPNFFIDRPIFAWVIAIIIMLAGGLAILKLPVAQYPTIAPPAVTISA+YPGADAKTVQDT
E. coli 1     MPNFFIDRPIFAWVIAIIIMLAGGLAILKLPVAQYPTIAPPAVTISASYPGADAKTVQDT  60

S. ent  61    VTQVIEQNMNGIDNLMYMSSNSDSTGTVQITLTFESGTDADIAQVQVQNKLQLAMPLLPQ  120
              VTQVIEQNMNGIDNLMYMSSNSDSTGTVQITLTFESGTDADIAQVQVQNKLQLAMPLLPQ
E. coli 61    VTQVIEQNMNGIDNLMYMSSNSDSTGTVQITLTFESGTDADIAQVQVQNKLQLAMPLLPQ  120

S. ent  121   EVQQQGVSVEKSSSSFLMVVGVINTDGTMTQEDISDYVAANMKDPISRTSGVGDVQLFGS  180
              EVQQQGVSVEKSSSSFLMVVGVINTDGTMTQEDISDYVAANMKD ISRTSGVGDVQLFGS
E. coli 121   EVQQQGVSVEKSSSSFLMVVGVINTDGTMTQEDISDYVAANMKDAISRTSGVGDVQLFGS  180

S. ent  181   QYAMRIWMNPTELTKYQLTPVDVINAIKAQNAQVAAGQLGGTPPVKGQQLNASIIAQTRL  240
              QYAMRIWMNP EL K+QLTPVDVI AIKAQNAQVAAGQLGGTPPVKGQQLNASIIAQTRL
E. coli 181   QYAMRIWMNPNELNKFQLTPVDVITAIKAQNAQVAAGQLGGTPPVKGQQLNASIIAQTRL  240

S. ent  241   TSTDEFGKILLKVNQDGSQVRLRDVAKIELGGENYDVIAKFNGQPASGLGIKLATGANAL  300
              TST+EFGKILLKVNQDGS+V LRDVAKIELGGENYD+IA+FNGQPASGLGIKLATGANAL
E. coli 241   TSTEEFGKILLKVNQDGSRVLLRDVAKIELGGENYDIIAEFNGQPASGLGIKLATGANAL  300

S. ent  301   DTATAIRAELKKMEPFFPPGMKIVYPYDTTPFVKISIHEVVKTLVEAIILVFLVMYLFLQ  360
              DTA AIRAEL KMEPFFP G+KIVYPYDTTPFVKISIHEVVKTLVEAIILVFLVMYLFLQ
E. coli 301   DTAAAIRAELAKMEPFFPSGLKIVYPYDTTPFVKISIHEVVKTLVEAIILVFLVMYLFLQ  360

S. ent  361   NFRATLIPTIAVPVVLLGTFAVLAAFGFSINTLTMFGMVLAIGLLVDDAIVVVENVERVM  420
              NFRATLIPTIAVPVVLLGTFAVLAAFGFSINTLTMFGMVLAIGLLVDDAIVVVENVERVM
E. coli 361   NFRATLIPTIAVPVVLLGTFAVLAAFGFSINTLTMFGMVLAIGLLVDDAIVVVENVERVM  420

S. ent  421   TEEGLPPKEATRKSMGQIQGALVGIAMVLSAIFIPMAFFGGSTGAIYRQFSITIVSAMAL  480
               EEGLPPKEATRKSMGQIQGALVGIAMVLSA+F+PMAFFGGSTGAIYRQFSITIVSAMAL
E. coli 421   AEEGLPPKEATRKSMGQIQGALVGIAMVLSAVFVPMAFFGGSTGAIYRQFSITIVSAMAL  480

S. ent  481   SVLVALILTPALCATMLKPVAKGDHGEGKKGFFGWFNRLFDKSTHHYTDSVGNILRSTGR  540
              SVLVALILTPALCATMLKP+AKGDHGEGKKGFFGWFNR+F+KSTHHYTDSVG ILRSTGR
E. coli 481   SVLVALILTPALCATMLKPIAKGDHGEGKKGFFGWFNRMFEKSTHHYTDSVGGILRSTGR  540

S. ent  541   YLLLYLIIVVGMAYLFVRLPSSFLPDEDQGVFLTMVQLPAGATQERTQKVLDEVTDYYLN  600
              YL+LYLIIVVGMAYLFVRLPSSFLPDEDQGVF+TMVQLPAGATQERTQKVL+EVT YYL
E. coli 541   YLVLYLIIVVGMAYLFVRLPSSFLPDEDQGVFMTMVQLPAGATQERTQKVLNEVTHYYLT  600

S. ent  601   KEKANVESVFAVNGFGFAGRGQNTGIAFVSLKDWADRPGEKNKVEAITQRATAAFSQIKD  660
              KEK NVESVFAVNGFGFAGRGQNTGIAFVSLKDWADRPGE+NKVEAIT RAT AFSQIKD
E. coli 601   KEKNNVESVFAVNGFGFAGRGQNTGIAFVSLKDWADRPGEENKVEAITMRATRAFSQIKD  660

S. ent  661   AMVFAFNLPAIVELGTATGFDFELIDQAGLGHEKLTQARNQLFGEVAKYPDLLVGVRPNG  720
              AMVFAFNLPAIVELGTATGFDFELIDQAGLGHEKLTQARNQL  E AK+PD+L  VRPNG
E. coli 661   AMVFAFNLPAIVELGTATGFDFELIDQAGLGHEKLTQARNQLLAEAAKHPDMLTSVRPNG  720

S. ent  721   LEDTPQFKIDIDQEKAQALGVSISDINTTLGAAWGGSYVNDFIDRGRVKKVYVMSEAKYR  780
              LEDTPQFKIDIDQEKAQALGVSI+DINTTLGAAWGGSYVNDFIDRGRVKKVYVMSEAKYR
E. coli 721   LEDTPQFKIDIDQEKAQALGVSINDINTTLGAAWGGSYVNDFIDRGRVKKVYVMSEAKYR  780

S. ent  781   MLPDDINDWYVRGSDGQMVPFSAFSSSRWEYGSPRLERYNGLPSMEILGQAAPGKSTGEA  840
              MLPDDI DWYVR +DGQMVPFSAFSSSRWEYGSPRLERYNGLPSMEILGQAAPGKSTGEA
E. coli 781   MLPDDIGDWYVRAADGQMVPFSAFSSSRWEYGSPRLERYNGLPSMEILGQAAPGKSTGEA  840

S. ent  841   MAMMEELASKLPSGIGYDWTGMSYQERLSGNQAPALYAISLIVVFLCLAALYESWSIPFS  900
              M +ME+LASKLP+G+GYDWTGMSYQERLSGNQAP+LYAISLIVVFLCLAALYESWSIPFS
E. coli 841   MELMEQLASKLPTGVGYDWTGMSYQERLSGNQAPSLYAISLIVVFLCLAALYESWSIPFS  900

S. ent  901   VMLVVPLGVIGALLAATFRGLTNDVYFQVGLLTTIGLSAKNAILIVEFAKDLMDKEGKGL  960
              VMLVVPLGVIGALLAATFRGLTNDVYFQVGLLTTIGLSAKNAILIVEFAKDLMDKEGKGL
E. coli 901   VMLVVPLGVIGALLAATFRGLTNDVYFQVGLLTTIGLSAKNAILIVEFAKDLMDKEGKGL  960

S. ent  961   VEAMLEAVRMRLRPILMTSLAFMLGVMPLVISSGAGSGAQNAVGTGVLGGMVTATVLAIF  1020
              +EA L+AVRMRLRPILMTSLAF+LGVMPLVIS+GAGSGAQNAVGTGV+GGMVTATVLAIF
E. coli 961   IEATLDAVRMRLRPILMTSLAFILGVMPLVISTGAGSGAQNAVGTGVMGGMVTATVLAIF  1020

S. ent  1021  FVPVFFVVVRRRFSRKSEDIEHSHSTEHR  1049
              FVPVFFVVVRRRFSRK+EDIEHSH+ +H
E. coli 1021  FVPVFFVVVRRRFSRKNEDIEHSHTVDHH  1049
```

Figure S2. Sequence alignment of AcrB-Ec and AcrB-Sa. *Escherichia coli* AcrB from *Escherichia coli* str. K-12 substr. MG1655 (NCBI Reference Sequence: NP_414995.1) and AcrB from *Salmonella enterica* (NCBI Reference Sequence: WP_109350990.1). Alignment performed using BLAST. Mutations highlighted in yellow.

```
S. Typh 1     MPNFFIDRPIFAWVIAIIIMLAGGLAILKLPVAQYPTIAPPAVTISATYPGADAKTVQDT  60
              MPNFFIDRPIFAWVIAIIIMLAGGLAILKLPVAQYPTIAPPAVTISA+YPGADAKTVQDT
E. coli 1     MPNFFIDRPIFAWVIAIIIMLAGGLAILKLPVAQYPTIAPPAVTISASYPGADAKTVQDT  60

S. Typh 61    VTQVIEQNMNGIDNLMYMSSNSDSTGTVQITLTFESGTDADIAQVQVQNKLQLAMPLLPQ  120
              VTQVIEQNMNGIDNLMYMSSNSDSTGTVQITLTFESGTDADIAQVQVQNKLQLAMPLLPQ
E. coli 61    VTQVIEQNMNGIDNLMYMSSNSDSTGTVQITLTFESGTDADIAQVQVQNKLQLAMPLLPQ  120

S. Typh 121   EVQQQGVSVEKSSSSFLMVVGVINTDGMTQEDISDYVAANMKDPISRTSGVGDVQLFGS  180
              EVQQQGVSVEKSSSSFLMVVGVINTDGMTQEDISDYVAANMKD ISRTSGVGDVQLFGS
E. coli 121   EVQQQGVSVEKSSSSFLMVVGVINTDGMTQEDISDYVAANMKDAISRTSGVGDVQLFGS  180

S. Typh 181   QYAMRIWMNPTELTKYQLTPVDVINAIKAQNAQVAAGQLGGTPPVKGQQLNASIIAQTRL  240
              QYAMRIWMNP EL K+QLTPVDVI AIKAQNAQVAAGQLGGTPPVKGQQLNASIIAQTRL
E. coli 181   QYAMRIWMNPNELNKFQLTPVDVITAIKAQNAQVAAGQLGGTPPVKGQQLNASIIAQTRL  240

S. Typh 241   TSTDEFGKILLKVNQDGSQVRLRDVAKIELGGENYDVIAKFNGQPASGLGIKLATGANAL  300
              TST+EFGKILLKVNQDGS+V LRDVAKIELGGENYD+IA+FNGQPASGLGIKLATGANAL
E. coli 241   TSTEEFGKILLKVNQDGSRVLLRDVAKIELGGENYDIIAEFNGQPASGLGIKLATGANAL  300

S. Typh 301   DTATAIRAELKKMEPFFPPGMKIVYPYDTTPFVKISIHEVVKTLVEAIILVFLVMYLFLQ  360
              DTA AIRAEL KMEPFFP G+KIVYPYDTTPFVKISIHEVVKTLVEAIILVFLVMYLFLQ
E. coli 301   DTAAAIRAELAKMEPFFPSGLKIVYPYDTTPFVKISIHEVVKTLVEAIILVFLVMYLFLQ  360

S. Typh 361   NFRATLIPTIAVPVVLLGTFAVLAAFGFSINTLTMFGMVLAIGLLVDDAIVVVENVERVM  420
              NFRATLIPTIAVPVVLLGTFAVLAAFGFSINTLTMFGMVLAIGLLVDDAIVVVENVERVM
E. coli 361   NFRATLIPTIAVPVVLLGTFAVLAAFGFSINTLTMFGMVLAIGLLVDDAIVVVENVERVM  420

S. Typh 421   TEEGLPPKEATRKSMGQIQGALVGIAMVLSAVFIPMAFFGGSTGAIYRQFSITIVSAMAL  480
               EEGLPPKEATRKSMGQIQGALVGIAMVLSAVF+PMAFFGGSTGAIYRQFSITIVSAMAL
E. coli 421   AEEGLPPKEATRKSMGQIQGALVGIAMVLSAVFVPMAFFGGSTGAIYRQFSITIVSAMAL  480

S. Typh 481   SVLVALILTPALCATMLKPVAKGDHGEGKKGFFGWFNRLFDKSTHHYTDSVGNILRSTGR  540
              SVLVALILTPALCATMLKP+AKGDHGEGKKGFFGWFNR+F+KSTHHYTDSVG ILRSTGR
E. coli 481   SVLVALILTPALCATMLKPIAKGDHGEGKKGFFGWFNRMFEKSTHHYTDSVGGILRSTGR  540

S. Typh 541   YLLLYLIIVVGMAYLFVRLPSSFLPDEDQGVFLTMVQLPAGATQERTQKVLDEVTDYYLN  600
              YL+LYLIIVVGMAYLFVRLPSSFLPDEDQGVF+TMVQLPAGATQERTQKVL+EVT YYL
E. coli 541   YLVLYLIIVVGMAYLFVRLPSSFLPDEDQGVFMTMVQLPAGATQERTQKVLNEVTHYYLT  600

S. Typh 601   KEKANVESVFAVNGFGFAGRGQNTGIAFVSLKDWADRPGEKNKVEAITQRATAAFSQIKD  660
              KEK NVESVFAVNGFGFAGRGQNTGIAFVSLKDWADRPGE+NKVEAIT RAT AFSQIKD
E. coli 601   KEKNNVESVFAVNGFGFAGRGQNTGIAFVSLKDWADRPGEENKVEAITMRATRAFSQIKD  660

S. Typh 661   AMVFAFNLPAIVELGTATGFDFELIDQAGLGHEKLTQARNQLFGEVAKYPDLLVGVRPNG  720
              AMVFAFNLPAIVELGTATGFDFELIDQAGLGHEKLTQARNQL  E AK+PD+L  VRPNG
E. coli 661   AMVFAFNLPAIVELGTATGFDFELIDQAGLGHEKLTQARNQLLAEAAKHPDMLTSVRPNG  720

S. Typh 721   LEDTPQFKIDIDQEKAQALGVSISDINTTLGAAWGGSYVNDFIDRGRVKKVYVMSEAKYR  780
              LEDTPQFKIDIDQEKAQALGVSI+DINTTLGAAWGGSYVNDFIDRGRVKKVYVMSEAKYR
E. coli 721   LEDTPQFKIDIDQEKAQALGVSINDINTTLGAAWGGSYVNDFIDRGRVKKVYVMSEAKYR  780

S. Typh 781   MLPDDINDWYVRGSDGQMVPFSAFSSSRWEYGSPRLERYNGLPSMEILGQAAPGKSTGEA  840
              MLPDDI DWYVR +DGQMVPFSAFSSSRWEYGSPRLERYNGLPSMEILGQAAPGKSTGEA
E. coli 781   MLPDDIGDWYVRAADGQMVPFSAFSSSRWEYGSPRLERYNGLPSMEILGQAAPGKSTGEA  840

S. Typh 841   MAMMEELASKLPSGIGYDWTGMSYQERLSGNQAPALYAISLIVVFLCLAALYESWSIPFS  900
              M +ME+LASKLP+G+GYDWTGMSYQERLSGNQAP+LYAISLIVVFLCLAALYESWSIPFS
E. coli 841   MELMEQLASKLPTGVGYDWTGMSYQERLSGNQAPSLYAISLIVVFLCLAALYESWSIPFS  900

S. Typh 901   VMLVVPLGVIGALLAATFRGLTNDVYFQVGLLTTIGLSAKNAILIVEFAKDLMDKEGKGL  960
              VMLVVPLGVIGALLAATFRGLTNDVYFQVGLLTTIGLSAKNAILIVEFAKDLMDKEGKGL
E. coli 901   VMLVVPLGVIGALLAATFRGLTNDVYFQVGLLTTIGLSAKNAILIVEFAKDLMDKEGKGL  960

S. Typh 961   VEAMLEAVRMRLRPILMTSLAFMLGVMPLVISSGAGSGAQNAVGTGVLGGMVTATVLAIF  1020
              +EA L+AVRMRLRPILMTSLAF+LGVMPLVIS+GAGSGAQNAVGTGV+GGMVTATVLAIF
E. coli 961   IEATLDAVRMRLRPILMTSLAFILGVMPLVISTGAGSGAQNAVGTGVMGGMVTATVLAIF  1020

S. Typh 1021  FVPVFFVVVRRRFSRKSEDIEHSHSTEHR  1049
              FVPVFFVVVRRRFSRK+EDIEHSH+ +H
E. coli 1021  FVPVFFVVVRRRFSRKNEDIEHSHTVDHH  1049
```

Figure S3. Sequence alignment of AcrB-Ec and AcrB-Sa. *Escherichia coli* AcrB from *Escherichia coli* str. K-12 substr. MG1655 (NCBI Reference Sequence: NP_414995.1) and AcrB from *Salmonella enterica* subsp. *enterica* serovar Typhi (NCBI Reference Sequence: WP_109350990.1). Alignment performed using BLAST.

```
S. Para 1     MPNFFIDRPIFAWVIAIIIMLAGGLAIFKLPVAQYPTIAPPAVTISATYPGADAKTVQDT  60
              MPNFFIDRPIFAWVIAIIIMLAGGLAI KLPVAQYPTIAPPAVTISA+YPGADAKTVQDT
E. coli 1     MPNFFIDRPIFAWVIAIIIMLAGGLAILKLPVAQYPTIAPPAVTISASYPGADAKTVQDT  60

S. Para 61    VTQVIEQNMNGIDNLMYMSSNSDSTGTVQITLTFESGTDADIAQVQVQNKLQLAMPLLPQ  120
              VTQVIEQNMNGIDNLMYMSSNSDSTGTVQITLTFESGTDADIAQVQVQNKLQLAMPLLPQ
E. coli 61    VTQVIEQNMNGIDNLMYMSSNSDSTGTVQITLTFESGTDADIAQVQVQNKLQLAMPLLPQ  120

S. Para 121   EVQQQGVSVEKSSSSFLMVVGVINTDGTMTQEDISDYVAANMKDPISRTSGVGDVQLFGS  180
              EVQQQGVSVEKSSSSFLMVVGVINTDGTMTQEDISDYVAANMKD ISRTSGVGDVQLFGS
E. coli 121   EVQQQGVSVEKSSSSFLMVVGVINTDGTMTQEDISDYVAANMKDAISRTSGVGDVQLFGS  180

S. Para 181   QYAMRIWMNPTELTKYQLTPVDVINAIKAQNAQVAAGQLGGTPPVKGQQLNASIIAQTRL  240
              QYAMRIWMNP EL K+QLTPVDVI AIKAQNAQVAAGQLGGTPPVKGQQLNASIIAQTRL
E. coli 181   QYAMRIWMNPNELNKFQLTPVDVITAIKAQNAQVAAGQLGGTPPVKGQQLNASIIAQTRL  240

S. Para 241   TSTDEFGKILLKVNQDGSQVRLRDVAKIELGGENYDVIAKFNGQPASGLGIKLATGANAL  300
              TST+EFGKILLKVNQDGS+V LRDVAKIELGGENYD+IA+FNGQPASGLGIKLATGANAL
E. coli 241   TSTEEFGKILLKVNQDGSRVLLRDVAKIELGGENYDIIAEFNGQPASGLGIKLATGANAL  300

S. Para 301   DTATAIRAELKKMEPFFPPGMKIVYPYDTTPFVKISIHEVVKTLVEAIILVFLVMYLFLQ  360
              DTA AIRAEL KMEPFFP G+KIVYPYDTTPFVKISIHEVVKTLVEAIILVFLVMYLFLQ
E. coli 301   DTAAAIRAELAKMEPFFPSGLKIVYPYDTTPFVKISIHEVVKTLVEAIILVFLVMYLFLQ  360

S. Para 361   NFRATLIPTIAVPVVLLGTFAVLAAFGFSINTLTMFGMVLAIGLLVDDAIVVVENVERVM  420
              NFRATLIPTIAVPVVLLGTFAVLAAFGFSINTLTMFGMVLAIGLLVDDAIVVVENVERVM
E. coli 361   NFRATLIPTIAVPVVLLGTFAVLAAFGFSINTLTMFGMVLAIGLLVDDAIVVVENVERVM  420

S. Para 421   TEEGLPPKEATRKSMGQIQGALVGIAMVLSAVFIPMAFFGGSTGAIYRQFSITIVSAMAL  480
               EEGLPPKEATRKSMGQIQGALVGIAMVLSAVF+PMAFFGGSTGAIYRQFSITIVSAMAL
E. coli 421   AEEGLPPKEATRKSMGQIQGALVGIAMVLSAVFVPMAFFGGSTGAIYRQFSITIVSAMAL  480

S. Para 481   SVLVALILTPALCATMLKPVAKGDHGEGKKGFFGWFNRLFDKSTHHYTDSVGNILRSTGR  540
              SVLVALILTPALCATMLKP+AKGDHGEGKKGFFGWFNR+F+KSTHHYTDSVG ILRSTGR
E. coli 481   SVLVALILTPALCATMLKPIAKGDHGEGKKGFFGWFNRMFEKSTHHYTDSVGGILRSTGR  540

S. Para 541   YLLLYLIIVVGMAYLFVRLPSSFLPDADQGVFLTMVQLPAGATQERTQKVLDEVTDYYLN  600
              YL+LYLIIVVGMAYLFVRLPSSFLPD DQGVF+TMVQLPAGATQERTQKVL+EVT YYL
E. coli 541   YLVLYLIIVVGMAYLFVRLPSSFLPDEDQGVFMTMVQLPAGATQERTQKVLNEVTHYYLT  600

S. Para 601   KEKANVESVFAVNGFGFAGRGQNTGIAFVSLKDWADRPGEKNKVEAITQRATAAFSQIKD  660
              KEK NVESVFAVNGFGFAGRGQNTGIAFVSLKDWADRPGE+NKVEAIT RAT AFSQIKD
E. coli 601   KEKNNVESVFAVNGFGFAGRGQNTGIAFVSLKDWADRPGEENKVEAITMRATRAFSQIKD  660

S. Para 661   AMVFAFNLPAIVELGTATGFDFELIDQAGLGHEKLTQARNQLFGEVAKYPDLLVGVRPNG  720
              AMVFAFNLPAIVELGTATGFDFELIDQAGLGHEKLTQARNQL  E AK+PD+L  VRPNG
E. coli 661   AMVFAFNLPAIVELGTATGFDFELIDQAGLGHEKLTQARNQLLAEAAKHPDMLTSVRPNG  720

S. Para 721   LEDTPQFKIDIDQEKAQALGVSISDINTTLGAAWGGSYVNDFIDRGRVKKVYVMSEAKYR  780
              LEDTPQFKIDIDQEKAQALGVSI+DINTTLGAAWGGSYVNDFIDRGRVKKVYVMSEAKYR
E. coli 721   LEDTPQFKIDIDQEKAQALGVSINDINTTLGAAWGGSYVNDFIDRGRVKKVYVMSEAKYR  780

S. Para 781   MLPDDINDWYVRGSDGQMVPFSAFSSSRWEYGSPRLERYNGLPSMEILGQAAPGKSTGEA  840
              MLPDDI DWYVR +DGQMVPFSAFSSSRWEYGSPRLERYNGLPSMEILGQAAPGKSTGEA
E. coli 781   MLPDDIGDWYVRAADGQMVPFSAFSSSRWEYGSPRLERYNGLPSMEILGQAAPGKSTGEA  840

S. Para 841   MAMMEELASKLPSGIGYDWTGMSYQERLSGNQAPALYAISLIVVFLCLAALYESWSIPFS  900
              M +ME+LASKLP+G+GYDWTGMSYQERLSGNQAP+LYAISLIVVFLCLAALYESWSIPFS
E. coli 841   MELMEQLASKLPTGVGYDWTGMSYQERLSGNQAPSLYAISLIVVFLCLAALYESWSIPFS  900

S. Para 901   VMLVVPLGVIGALLAATFRGLTNDVYFQVGLLTTIGLSAKNAILIVEFAKDLMDKEGKGL  960
              VMLVVPLGVIGALLAATFRGLTNDVYFQVGLLTTIGLSAKNAILIVEFAKDLMDKEGKGL
E. coli 901   VMLVVPLGVIGALLAATFRGLTNDVYFQVGLLTTIGLSAKNAILIVEFAKDLMDKEGKGL  960

S. Para 961   VEATLEAVRMRLRPILMTSLAFMLGVMPLVISSGAGSGAQNAVGTGVLGGMVTATVLAIF  1020
              +EATL+AVRMRLRPILMTSLAF+LGVMPLVIS+GAGSGAQNAVGTGV+GGMVTATVLAIF
E. coli 961   IEATLDAVRMRLRPILMTSLAFILGVMPLVISTGAGSGAQNAVGTGVMGGMVTATVLAIF  1020

S. Para 1021  FVPVFFVVVRRRFSRKSEDIEHSHSTEHR  1049
              FVPVFFVVVRRRFSRK+EDIEHSH+ +H
E. coli 1021  FVPVFFVVVRRRFSRKNEDIEHSHTVDHH  1049
```

Figure S4. Sequence alignment of AcrB-Ec and AcrB-Sa. *Escherichia coli* AcrB from *Escherichia coli* str. K-12 substr. MG1655 (NCBI Reference Sequence: NP_414995.1) and AcrB from *Salmonella* AcrBs from *Salmonella enterica* subsp. *enterica* serovar Paratyphi A (NCBI Reference Sequence: SUH78479.1). Alignment performed using BLAST.

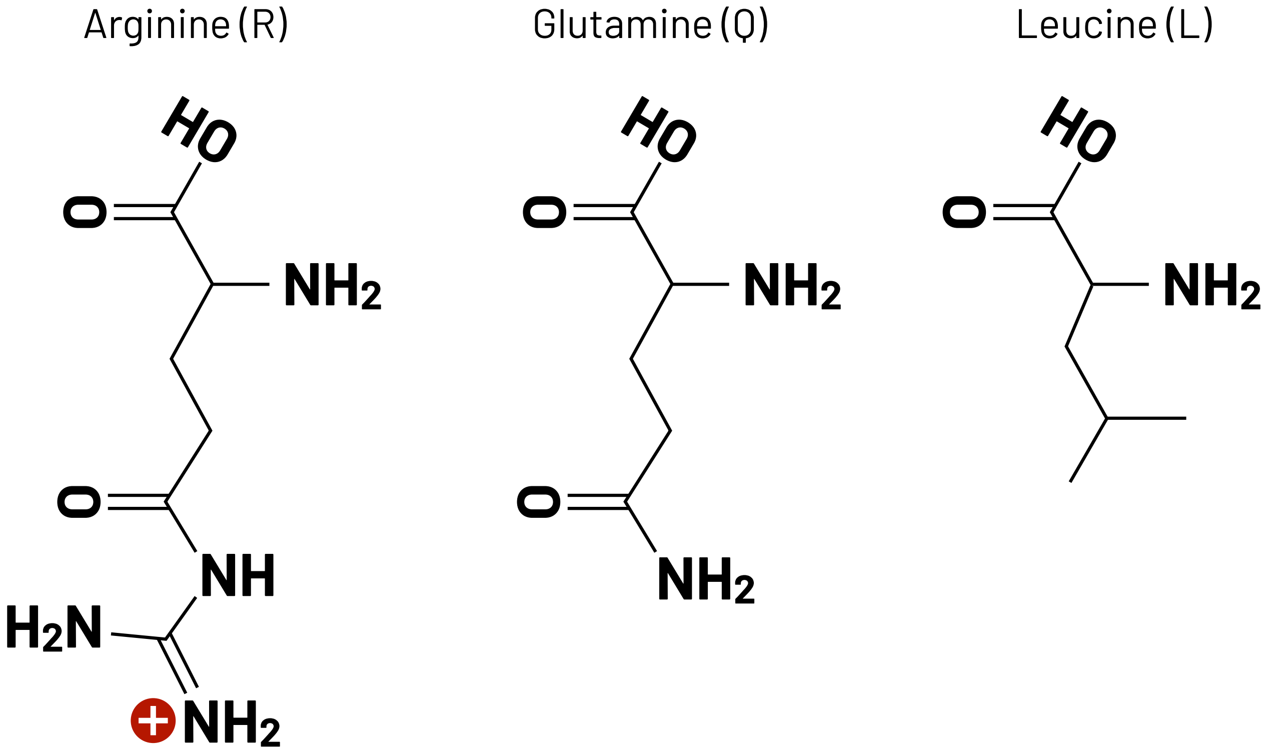

Figure S5. Simple amino acid comparison between Arg, Gln and Leu. Structural comparison between the three different amino acids found in wild-type and mutant strains within the AcrB efflux pump. Arginine is an amino acid with a positively charged and relatively long side chain. Glutamine has a polar, medium length side chain. Leucine has a hydrophobic and relatively short side chain.

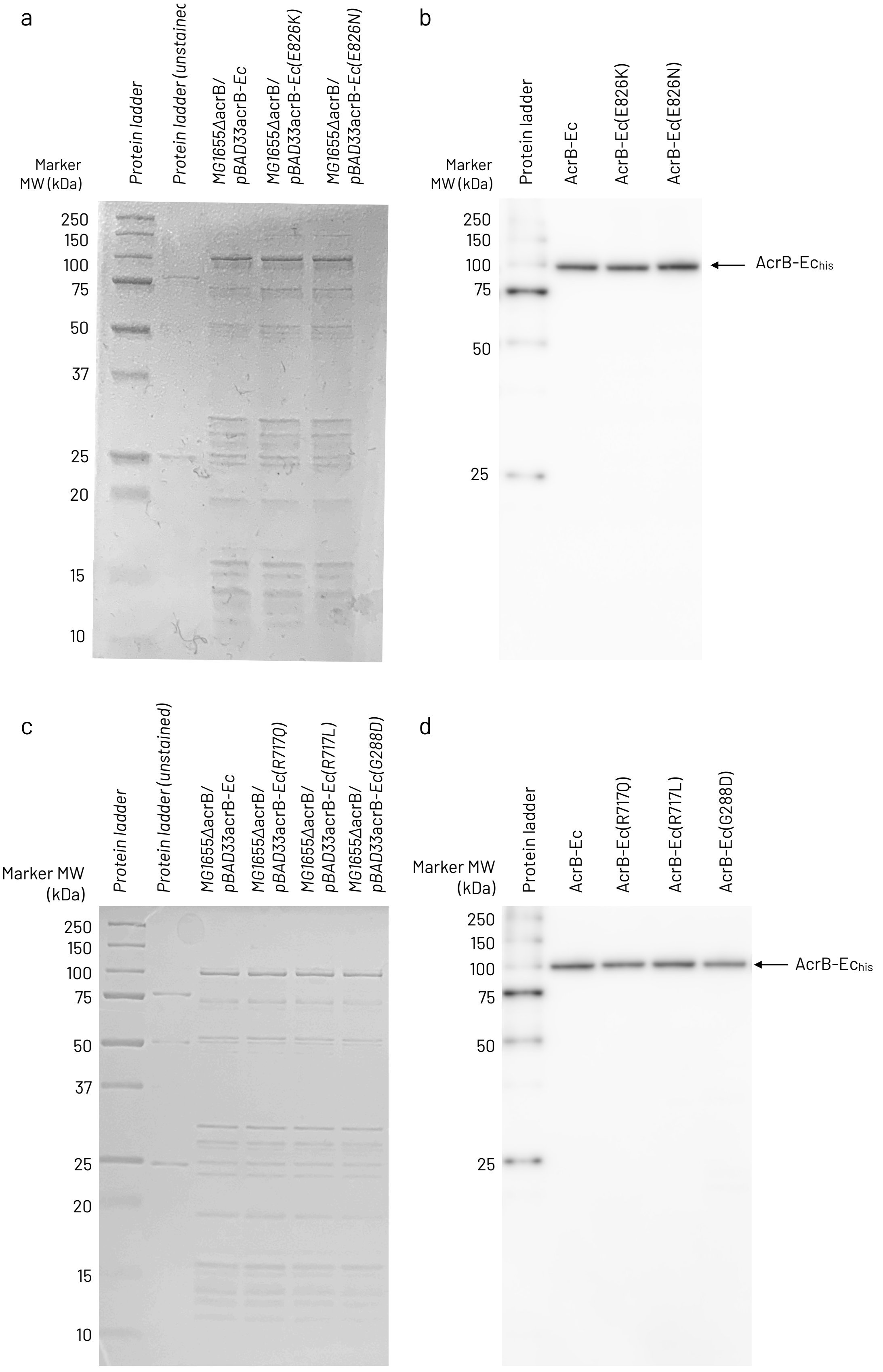

Figure S6. Expression of wild-type and mutant AcrB-Ec in MG1655Δ*acrB* cells. Western blotting analysis of wild-type and E826K, E826N, R717Q, R717L and G288D AcrB-Ec. (A,C) SDS-PAGE of the membrane fraction of the AcrB-Ec expressing cells. (B,D) Western Blotting analysis of the membrane fraction of AcrB-Ec expressing cells. Wild-type and mutant AcrB- Ec are expressed in similar amounts. Each well was loaded with 2 μg membrane fraction as determined by bicinchoninic acid (BCA) assay.

```
N. gono 1      MAKFFIDRPIFAWVISIFIIAAGIFGIKSLPVSQYPSVAAPTITLHAIYPGASAQVMEGS  60
               M  FFIDRPIFAWVI+I I+ AG   I  LPV+QYP++A P +T+ A YPGA A+ ++ +
E. coli 1      MPNFFIDRPIFAWVIAIIIMLAGGLAILKLPVAQYPTIAPPAVTISASYPGADAKTVQDT  60

N. gono 61     VLSVIERNMNGVEGLDYMSTSADSSGSGSVSLTFTPDTDENLAQVEVQNKLSEVLSTLPA  120
               V  VIE+NMNG++ L YMS+++DS+G+  ++LTF   TD ++AQV+VQNKL   +  LP
E. coli 61     VTQVIEQNMNGIDNLMYMSSNSDSTGTVQITLTFESGTDADIAQVQVQNKLQLAMPLLPQ  120

N. gono 121    TVQQYGVTVSKARSNFLMIV-MLSSD-VQSTEEMNDYAQRNVVPELQRIEGVGQVRLFGA  178
                VQQ GV+V K+ S+FLM+V ++++D   + E+++DY   N+   + R  GVG V+LFG+
E. coli 121    EVQQQGVSVEKSSSSFLMVVGVINTDGTMTQEDISDYVAANMKDAISRTSGVGDVQLFGS  180

N. gono 179    QRAMRIWVDPKKLQNYNLSFADVGSALSAQNIQISAGSIGSLPAVRGQTVTATVTAQGQL  238
               Q AMRIW++P +L  + L+  DV +A+ AQN Q++AG +G  P V+GQ + A++ AQ +L
E. coli 181    QYAMRIWMNPNELNKFQLTPVDVITAIKAQNAQVAAGQLGGTPPVKGQQLNASIIAQTRL  240

N. gono 239    GTAEEFGNVILRANTDGSNIYLKDVAKVGLGMEDYSSSTRLNGVNTTGMAVMLSNSGNAM  298
                + EEFG ++L+ N DGS + L+DVAK+ LG E+Y      NG   +G+ + L+   NA+
E. coli 241    TSTEEFGKILLKVNQDGSRVLLRDVAKIELGGENYDIIAEFNGQPASGLGIKLATGANAL  300

N. gono 299    ATAKAVKERLAVLEKYFPQGMSWKTPYDTSKFVEISIEKVIHTLIEAMVLVFVVMYLFLQ  358
                TA A++  LA +E +FP G+    PYDT+ FV+ISI +V+ TL+EA++LVF+VMYLFLQ
E. coli 301    DTAAAIRAELAKMEPFFPSGLKIVYPYDTTPFVKISIHEVVKTLVEAIILVFLVMYLFLQ  360

N. gono 359    NIRYTLIPTIVVPISLLGGFAFISYMGMSINVLTMFAMTLVIGIVVDDAIVVVENVERIM  418
               N R TLIPTI VP+ LLG FA ++  G SIN LTMF M L IG++VDDAIVVVENVER+M
E. coli 361    NFRATLIPTIAVPVVLLGTFAVLAAFGFSINTLTMFGMVLAIGLLVDDAIVVVENVERVM  420

N. gono 419    AGEGLPPKEATKKAMGQISGAVIGITAVLISVFVPLAMFSGAAGNIYKQFALTMASSIAF  478
               A EGLPPKEAT+K+MGQI GA++GI  VL +VFVP+A F G+ G IY+QF++T+ S++A
E. coli 421    AEEGLPPKEATRKSMGQIQGALVGIAMVLSAVFVPMAFFGGSTGAIYRQFSITIVSAMAL  480

N. gono 479    SAFLALTLTPALCATMLKTIPKGHH-EEKKGFFGWFNKKFDSWTHGYEGRVAKVLRKTFR  537
               S  +AL LTPALCATMLK I KG H E KKGFFGWFN+ F+  TH Y   V  +LR T R
E. coli 481    SVLVALILTPALCATMLKPIAKGDHGEGKKGFFGWFNRMFEKSTHHYTDSVGGILRSTGR  540

N. gono 538    MMVVYIGLAVVGVFLFMRLPTSFLPTEDQGFVMVSVQLPAGATKERTDATLAQVTQ--LA  595
                +V+Y+ + V   +LF+RLP+SFLP EDQG  M  VQLPAGAT+ERT   L +VT   L
E. coli 541    YLVLYLIIVVGMAYLFVRLPSSFLPDEDQGVFMTMVQLPAGATQERTQKVLNEVTHYYLT  600

N. gono 596    KSIPEIENIITVSGFSFSGSGQNMAMGFAILKDWNERTASGSDAVAVAGKLTGMMMGTLK  655
               K    +E++  V+GF F+G GQN  + F  LKDW +R    +   A+  + T      +K
E. coli 601    KEKNNVESVFAVNGFGFAGRGQNTGIAFVSLKDWADRPGEENKVEAITMRAT-RAFSQIK  659

N. gono 656    DGFGIAVVPPPILELGNGSGLSINLQDRNNTGHTALLAKRNELIQKMRASGLFDP---ST  712
               D    A   P I+ELG  +G    L D+   GH  L   RN+L+    A     P   ++
E. coli 660    DAMVFAFNLPAIVELGTATGFDFELIDQAGLGHEKLTQARNQLL----AEAAKHPDMLTS  715

N. gono 713    VRAGGLEDSPQLKIDINRAAAAAQGISFADIRTALASALSSSYVSDFPNQGRLQRVMVQA  772
               VR  GLED+PQ KIDI++  A A G+S  DI T L +A   SYV+DF ++GR+++V V +
E. coli 716    VRPNGLEDTPQFKIDIDQEKAQALGVSINDINTTLGAAWGGSYVNDFIDRGRVKKVYVMS  775

N. gono 773    DEDARMQPADILNLTVPNKSGVAVPLSTIATVSWENGTEQSVRFNGYPSMKLSASPATGV  832
               +   RM P DI +  V    G  VP S  ++  WE G+ +  R+NG PSM++    A G
E. coli 776    EAKYRMLPDDIGDWYVRAADGQMVPFSAFSSSRWEYGSPRLERYNGLPSMEILGQAAPGK  835

N. gono 833    STGQAMAAVQKMVDELGGGYSLEWGGQSREEAKGGSQTLILYGLAVAAVFLVLAALYESW  892
               STG+AM  ++++  +L  G   +W G S +E   G+Q   LY +++  VFL LAALYESW
E. coli 836    STGEAMELMEQLASKLPTGVGYDWTGMSYQERLSGNQAPSLYAISLIVVFLCLAALYESW  895

N. gono 893    SIPLAVILVIPLGLIGAAAGVTGRNLFEGLLGSVPSFANDIYFQVGFVTVMGLSAKNAIL  952
              SIP +V+LV+PLG+IGA    T R L            ND+YFQVG +T +GLSAKNAIL
E. coli 896    SIPFSVMLVVPLGVIGALLAATFRGL-----------TNDVYFQVGLLTTIGLSAKNAIL  944

N. gono 953    IIEFAKDL-QAQGKSAVEAALEAARLRFRPIIMTSFAFILGVVPLYIAAGASSASQRAIG  1011
               I+EFAKDL   +GK  +EA L+A R+R RPI+MTS AFILGV+PL I+ GA S +Q A+G
E. coli 945    IVEFAKDLMDKEGKGLIEATLDAVRMRLRPILMTSLAFILGVMPLVISTGAGSGAQNAVG  1004

N. gono 1012   TTVFWGMLIGTLLSV  1026
               T V  GM+  T+L++
E. coli 1005   TGVMGGMVTATVLAI  1019
```

Figure S7. Sequence alignment of AcrB and MtrD. *Escherichia coli* AcrB from *E. coli* str. K-12 substr. MG1655 (NCBI Reference Sequence: NP_414995.1) and MtrD from *Neisseria gonorrhoeae* (NCBI Reference Sequence: WP_162503037.1). Alignment performed using BLAST. AcrB E826 and MtrD K823 are highlighted in yellow.
